# mTORC2 and AMPK differentially regulate muscle triglyceride content via Perilipin 3

**DOI:** 10.1016/j.molmet.2016.06.007

**Published:** 2016-06-22

**Authors:** Maximilian Kleinert, Benjamin L. Parker, Rima Chaudhuri, Daniel J. Fazakerley, Annette Serup, Kristen C. Thomas, James R. Krycer, Lykke Sylow, Andreas M. Fritzen, Nolan J. Hoffman, Jacob Jeppesen, Peter Schjerling, Markus A. Ruegg, Bente Kiens, David E. James, Erik A. Richter

**Affiliations:** 1 Section of Molecular Physiology, Department of Nutrition, Exercise and Sports, Faculty of Science, University of Copenhagen, Copenhagen, Denmark; 2 Charles Perkins Centre, School of Molecular Bioscience, The University of Sydney, Sydney, Australia; 3 Diabetes and Metabolism Division, Garvan Institute of Medical Research, Darlinghurst, Australia; 4 Institute of Sports Medicine, Department of Orthopedic Surgery M, Bispebjerg Hospital and Center for Healthy Aging, Faculty of Health and Medical Sciences, University of Copenhagen, Copenhagen, Denmark; 5 Biozentrum, University of Basel, Basel, Switzerland; 6 Sydney Medical School, The University of Sydney, Sydney, Australia; 7 Institute for Diabetes and Obesity, Helmholtz Zentrum München, German Research Center for Environmental Health, Neuherberg, Germany

**Keywords:** PLIN3, RICTOR, mTOR, Metabolism, Akt

## Abstract

**Objective:**

We have recently shown that acute inhibition of both mTOR complexes (mTORC1 and mTORC2) increases whole-body lipid utilization, while mTORC1 inhibition had no effect. Therefore, we tested the hypothesis that mTORC2 regulates lipid metabolism in skeletal muscle.

**Methods:**

Body composition, substrate utilization and muscle lipid storage were measured in mice lacking mTORC2 activity in skeletal muscle (specific knockout of RICTOR (Ric mKO)). We further examined the RICTOR/mTORC2-controlled muscle metabolome and proteome; and performed follow-up studies in other genetic mouse models and in cell culture.

**Results:**

Ric mKO mice exhibited a greater reliance on fat as an energy substrate, a re-partitioning of lean to fat mass and an increase in intramyocellular triglyceride (IMTG) content, along with increases in several lipid metabolites in muscle. Unbiased proteomics revealed an increase in the expression of the lipid droplet binding protein Perilipin 3 (PLIN3) in muscle from Ric mKO mice. This was associated with increased AMPK activity in Ric mKO muscle. Reducing AMPK kinase activity decreased muscle PLIN3 expression and IMTG content. AMPK agonism, in turn, increased PLIN3 expression in a FoxO1 dependent manner. PLIN3 overexpression was sufficient to increase triglyceride content in muscle cells.

**Conclusions:**

We identified a novel link between mTORC2 and PLIN3, which regulates lipid storage in muscle. While mTORC2 is a negative regulator, we further identified AMPK as a positive regulator of PLIN3, which impacts whole-body substrate utilization and nutrient partitioning.

## Highlights

• Lack of mTORC2 activity in muscle alters overall body composition, substrate utilization and the muscle metabolite profile.
• mTORC2 and AMPK regulate IMTG and PLIN3 in opposite fashion in muscle.
• AMPK agonism increases PLIN3 expression in a FoxO1 dependent manner.
• PLIN3 overexpression increases triglyceride levels in muscle cells.

## Graphical abstract

**Figure:**
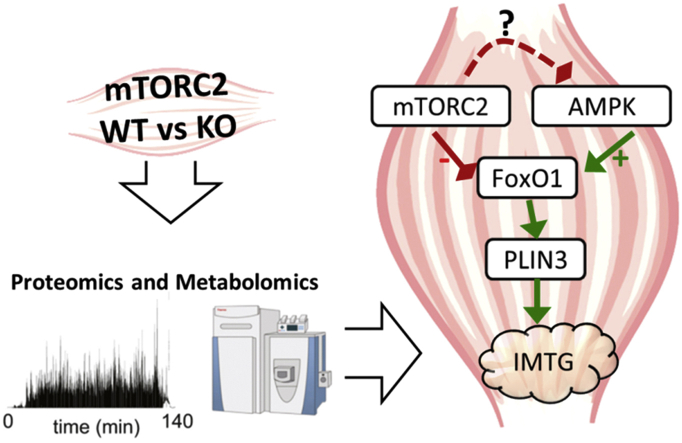

## 1 Introduction

The mammalian target of rapamycin (mTOR) is a serine/threonine protein kinase that is found in two distinct mTOR complexes, mTOR complex 1 (mTORC1) and mTORC2. RAPTOR (regulatory-associated protein of mTOR) is the defining subunit of mTORC1, which is a vital regulator of cell growth. The subunits RICTOR (rapamycin-insensitive companion of mTOR) and SIN1 (SAPK-interacting protein 1) are unique to mTORC2. Growth factors activate mTORC2 [31], [45], and its substrates include Akt, SGK1, and PKCα. As a kinase for Akt Ser473 phosphorylation [34], mTORC2 is positioned in the canonical insulin signaling pathway responsible for regulating glucose uptake. Therefore, it is not surprising that in adipose tissue [11], [25] and in liver [14], [26], [43] mTORC2 is required for maintaining glucose homeostasis. Similarly, evidence from cultured muscle cells [42] and incubated mouse skeletal muscles [24] implicated mTORC2 as a regulator of muscle glucose metabolism. In agreement, muscle specific RICTOR knockout (Ric mKO) mice are glucose intolerant [24]. Moreover, we recently demonstrated that mTORC2 is necessary for normal insulin-stimulated muscle glucose uptake *in vivo* [22] and others have suggested that mTORC2 is required for adrenergic stimulation of glucose uptake in skeletal muscle [35]. Despite its position upstream of Akt within the insulin signaling network, questions remain about how mTORC2 signaling impacts glucose metabolism specifically in muscle, because Akt signaling to substrates appears to be normal in muscle lacking mTORC2 activity [3], [22], [35]. For example, in response to a physiological insulin stimulation *in vivo*, phosphorylation of AS160, a well described Akt substrate and key regulator of glucose uptake, was not inhibited, but hyper-phosphorylated in muscle lacking RICTOR, despite a robust reduction in Akt Ser473 phosphorylation [22] in Ric mKO muscle.

Since muscle is an important tissue for whole-body glucose homeostasis, especially in humans [12], [20], understanding mTORC2-mediated regulation of energy metabolism may reveal new therapeutic strategies for treating diseases that are characterized by dysregulated glucose metabolism (e.g. type 2 diabetes). We have recently shown that acute inhibition of both mTOR complexes increases whole-body lipid utilization in mice, while mTORC1 inhibition had no effect [22]. We therefore hypothesized that, in addition to its role in glucose metabolism, mTORC2 regulates lipid metabolism. In agreement with our hypothesis, we found evidence for mTORC2 regulation of muscle lipid metabolism, specifically lipid storage, which appears to be linked to specific changes in the mTORC2-controlled proteome.

## 2 Material and methods

### 2.1 Animals

Female muscle-specific RICTOR knockout (Ric mKO: RICTOR^floxed/floxed^, HSA-Cre^−/+^) and WT (Ric WT: RICTOR^floxed/floxed^, HSA-Cre^−/−^) littermates aged 9–16 weeks were used [3]. Female C57Bl/6 mice over-expressing a muscle-specific, kinase-dead AMPKα_2_ construct (AMPK KD) and corresponding WT littermates [30], aged 12–21 weeks were used. Muscle-specific FoxO1 knockout muscle samples (FoxO1 mKO) on a C57BL/6 background were a gift from Dr. Febbraio, Baker Heart and Diabetes Institute, Melbourne, Australia. Mice were group-housed when possible, kept on a 12:12-h light–dark cycle, and had free access to standard rodent chow diet (Altromin no. 1324; Brogaarden, Denmark) and water. All experiments were approved by the Danish Animal Experimental Inspectorate.

### 2.2 qRT-PCR

*Ric mKO mice*: ∼20 mg quadriceps was homogenized in TRIzol (Life Technologies). Total RNA was isolated using 1-Bromo 3-Chloropropane for phase separation and isopropanol for total RNA precipitation according to manufacturer's instructions. RNA quantity and purity was determined by UV measurements using a Nanodrop ND-1000. cDNA was synthesized from all samples at the same time using the same batch of enzyme mix from 1 μg total RNA using reverse transcriptase and oligo-dT primers (Omniscript RT kit, Qiagen). The primer sequences are listed in the Supplementary data online. LightCycler 480 SYBR Green 1 Master Mix and LightCycler 480 (both Roche Applied Science) were used to quantify cDNA. The PCR cycle conditions were 94 °C for 10 min followed by 40 cycles at 94 °C for 15 s, 60 °C for 30 s, and 72 °C for 30 s. Assay efficiencies were checked using serial dilution of mouse cDNA, and only primers that fell in the 85–115% range were used. Gene expression levels were normalized to the house-keeping gene, EEF2 or HRPT, (no Ct difference between Ric WT and mKO) and made relative to the Ric WT condition using the ΔΔCt method. *AMPK KD mice*: Total RNA was isolated from ∼20 mg AMPK KD and wildtype quadriceps muscle with a modified guanidinium thiocyanate-phenol-chloroform extraction method adapted from Chomczynski and Sacchi [10], described previously by Pilegaard et al. [33], except that the tissue was homogenized for 3 min at 30 Hz in a TissueLyzerII (Qiagen, CA, USA). RNA concentration and purity was tested by Nanodrop (Nanodrop 1000, Thermo Scientific, MA, USA). 4 μg RNA was transcribed to cDNA by Superscript II RNase H^−^ system and Oligo dT (Invitrogen, USA) [33]. mRNA content was determined in triplicates by real-time PCR (ABI PRISM 7900 Sequence Detection Systems; Applied Biosystems) using the fluorogenic 5′ nuclease assay with TaqMan probes and universal mastermix with UNG using the following cycle profile: 50 °C for 2 min + 95 °C for 10 min + (95 °C for 15 s + 60 °C for 1 min) × 40. PLIN3 and β-actin probes and primers were pre-developed assay reagents from Applied Biosystems, USA (PLIN3 # mm04208646_g1 and β-actin # 4352341E). The total amount of PLIN3 and β-actin mRNA was determined by gene-specific standard curves made from a serial dilution of a pooled sample made from all samples. PLIN3 mRNA was normalized to total amount of β-actin mRNA, and there was no difference in β-actin mRNA between AMPK KD and wildtype. *L6 cells and C2C12 cells*: Total RNA was extracted from using TRIzol reagent (Invitrogen). cDNA synthesis was performed using PrimeScript 1st strand cDNA Synthesis Kit (Clontech, Takara Bio Company). PCR reactions were carried out using FastStart Universal SYBR Green Master (Roche) on the LightCycler 480 II (Roche). β-actin (Actb) or HRPT were used as internal controls and gene expression levels were normalized to Actb or HRPT and made relative to the control condition using the ΔΔCt method. Primers used in L6 cells: Plin3 F: 5′-CCTGATTGCCACATCTCCA-3′; Plin3 R: 5′-GCCCAACCTGACAAAGTAGC-3′; Actb F: 5′-CTGGCTCCTAGCACCATGA-3′; Actb R: 5′-ACTCCTGCTTGCTGATCCAC-3′; and FoxO1 F: 5′-GGATAAGGGCGACAGCAACA-3′; FoxO1 R: 5′-TTCCCACTCTTGCCTCCCT-3′. Primers used in C2C12 cells: HRPT: F: 5′-AAGCTTGCTGGTGAAAAGGA-3′; HRPT R: 5′-TTGCGCTCATCTTAGGCTTT-3′; Plin3 F: 5′-GCCCAAGAGATGGTGTCTAGC-3′; Plin3 R: 5′-CCGGTCACTACGGACTTTGT-3′. Their efficiency was tested.

### 2.3 Nuclear fractionation

As adapted from Senf et al. [37] with minor changes. ∼30 mg of quadriceps muscle from fed Ric mKO and AMPK KD mice were homogenized. The nuclear pellet was rotated for 2 h with 0.42 M NaCl. In pilot experiments efficient separation of the nuclear fraction from the cytosolic fraction was verified with β-actin (cytosolic marker) and lamin A/C (nuclear marker).

### 2.4 TG determinations

Muscle (quadriceps) and liver were freeze-dried and dissected free of all visible fat, blood and connective tissue and IMTG and liver TG content was assayed as previously described [21]. TG determinations in C2C12 myotubes were carried out as described in [36].

### 2.5 PLIN3 overexpression experiments

C2C12 cells were maintained in Dulbecco's modified Eagle's medium (DMEM) (Invitrogen) containing antibiotics and 10% FCS. To differentiate cells into myotubes, cells were maintained in DMEM (containing antibiotics) now supplemented with 2% HS (differentiation medium) for 5–6 days. PLIN3 overexpression was accomplished by Lipofectamine-2000 mediated transfection with a pCMV-Myc-C PLIN3 ORF clone. Control cells were transfected with a pCAG-GFP empty vector clone. To verify transfection myc-tag and GFP levels were assessed by standard WB technique and PLIN3 expression was assayed by qPCR 24 h post transfection. Other transfected myotubes were fixed with 4% paraformaldehyde and Dapi stained after 24 h. Staining intensity fluorescence was measured at 340/460 nM. Other transfected myotubes were incubated for 6 h or 18 h with either 750 μmol/L palmitic acid made up in differentiation medium containing 2% BSA (free of fatty acids) or for 18 h in differentiation medium only containing 2% BSA.

### 2.6 Western blot analysis

Tissues were homogenized with stainless steel pellets that shook 2 × 45 s at 30 Hz using a Tissuelyser II (Qiagen, USA) in ice-cold homogenization buffer (10% Glycerol, 20 mM Na-pyrophosphate, 150 mM NaCl, 50 mM HEPES (pH 7.5), 1% NP-40, 20 mM β-glycerophosphate, 10 mM NaF, 2 mM PMSF, 1 mM EDTA (pH 8.0), 1 mM, EGTA (pH 8.0), 10 μg/ml Aprotinin, 10 μg/ml Leupeptin, 2 mM Na3VO4, 3 mM Benzamidine, 5 mM Nicotinamide). Homogenates were rotated end-over-end for 1 h at 4 °C, and cleared by centrifugation at 13000 ×g for 20 min at 4 °C. Lysate protein content was determined by bicinchoninic acid method and lysates were diluted to the same protein concentration (1–2 μg/μl). Total protein and phosphorylation levels of indicated proteins were determined by standard immunoblotting, loading equal amounts of protein (5–40 ug).

### 2.7 Indirect calorimetry and total activity

Mice were acclimatized to indirect calorimetry cages (PhenoMaster, TSE, Germany) for 3 days, before expired carbon dioxide (VCO2) and inhaled oxygen (VO2) were measured over another 3 days to determine respiratory exchange ratio (RER). Total activity was measured with laser beam breaks and in a subset of mice food intake was measured.

### 2.8 Body composition

Mouse body composition was assessed by MRI-scan (EchoMRI-4 in 1TM for Live Animals, Echo Medical System LLC, Texas, USA) according to manufacturer's instructions.

### 2.9 L6 cell experiments

2-DG Experiments: L6 myoblasts (ATCC CRL-1458) were grown in growth media (α-MEM media (GIBCO #1257-063), 10% fetal bovine serum (GIBCO #26140-038), 100 units/mL penicillin, 100 μg/ml streptomycin, 0.25 μg/ml Fungizone^©^ (GIBCO #15240-062)) in 5% CO2, at 37 °C. For experiments, L6 myoblasts were seated in 6-well plates. Once confluent growth media was refreshed (2 ml) and interventions as indicated were added. 2-deoxyglucose (2-DG, Sigma) was made up as 2.5 and 0.25 M stock solution in distilled water and added to wells at 1:100 resulting in final concentration of 25 mM and 2.5 mM. Control wells received equal volumes of distilled water. A Compound C (Calbiochem) stock solution of 1 mM in 100% DMSO was diluted to 0.1 mM Compound C and 10% DMSO with distilled water and finally added to wells at 1:100 for a final concentration of 1 μM Compound C and 0.1% DMSO. Control wells received the same concentration and volume of DMSO. After 16 h cells were washed once with ice-cold PBS and lysed for WB: Cells were quickly dried, and scraped off in presence of 150 μl ice-cold cell lysis buffer (50 mM HEPES, 150 mM NaCl, 100 mM NaF, 10 mM sodium-pyrophosphate, 5 mM EDTA, 250 mM sucrose, 1 mM DTT, 1% (v/v) Triton X-100, 1 mM sodium orthovanadate, 1:100 protease inhibitor cocktail (#11697498001; Roche Applied Science; stock solution = 1 tablet in 0.5 ml distilled water), 5 mM Nicotinamide, pH 7.4). Cells were rigorously pipetted up and down 20 times and then centrifuged at 13000 ×g for 10 min at 4 °C. Supernatant (lysate) protein content was determined with the bicinchoninic acid (BCA) method using BSA standards (Pierce) and BCA assay reagents (Pierce) and all lysates were diluted to the same protein concentration (0.25–0.5 μg/μl). Total protein and phosphorylation levels of indicated proteins were determined by standard immunoblotting, loading equal amounts of protein (5–10 μg). AMPK activation experiments: L6 myoblasts were initially cultured as above, but without antibiotics. To differentiate myoblasts into myotubes fetal bovine serum concentration was reduced to 2%. Myotubes were used for experiments 5–9 days after the initiation of differentiation. To assess the effect of AMPK activation on PLIN3 expression, L6 cells were treated with 0.1% DMSO, 2 mM AICAR (Toronto Research Chemicals; in PBS) + 0.1%DMSO, 100 μM A-769662 (a kind gift from Dr. K. Sakamoto; in 0.1% DMSO) or both compounds. Cells were also treated with vehicle 10 μM Compound C where indicated (Calbiochem; in 0.1% DMSO). After 16 h cells were harvested for qPCR analysis (described elsewhere). Knockdown of FoxO1, and FoxO3 experiments: L6 myoblasts cultured as above, but without antibiotics, were transfected with scrambled siRNA, FOXO1-specific siRNA and/or FOXO3-specific siRNA using RNAiMax (Life Technologies) following the manufacturer's instructions. For knockdown of each FoxO isoform (1 and 3), cells were treated with a final concentration of 100 nM siRNA made up of equal amounts of a pool of three siRNAs for FOXO1 and two siRNA for FOXO3 (see sense sequences below). For simultaneous knockdown of both isoforms cells were treated with 100 nM of each siRNA pool (data of these experiments in Figure 5F–H). In separate experiments the effects of the individual FoxO1 siRNA sequences were assessed (results in Figure S3) essentially as above but with 100 nM of each siRNA. Assessment (by WB and qPCR) of the effect of AMPK activation on PLIN3 expression was performed as described above 36–48 h after transfection. Scramble, FoxO1 and FoxO3 siRNAs were from GenePharma. For WB analysis, cells were lysed in 2% SDS in PBS containing protease (Roche) and phosphatase inhibitors (1 mm sodium pyrophosphate, 2 mm sodium orthovanadate, 10 mm sodium fluoride) and protein content determination and immunoblotting was carried out as described above. qPCR analysis is described elsewhere. Knockdown of AMPKα: For siRNA knockdown of the AMPKα catalytic subunits, siRNA oligonucleotide sequences were purchased from Shanghai GenePharma Co., Ltd to target either rat AMPKα1 or rat AMPKα2. For all siRNA knockdown experiments rat L6 myoblasts were transfected at approximately 48 h post seeding (or ∼60% confluency). Calcium phosphate-based siRNA transfection was utilized for each well of L6 myoblasts seeded in a 6-well plate as follows, 3 μl of 20 μM Negative Control scrambled or 1.5 μl of each AMPKα siRNA oligonucleotide was added to 42.6 μl Buffer A (1 mM Tris/HCl, 0.1 mM EDTA, pH 7.9). After mixing, 2.4 μl 1M CaCl2 was added dropwise followed by another 12 μl 1M CaCl2. This siRNA mixture was then added dropwise to 60 μl Buffer B (50 mM HEPES pH 7.0, 280 mM NaCL, 0.75 mM Na2HPO4, 0.75 mM NaH2PO4). After 30 min incubation at room temperature, 120 μl of the combined siRNA mix was added dropwise to myoblasts in 6-well plates containing 1380 μl αMEM +10% FBS and incubated 12–16 h. Following 12–16 h incubation, media was aspirated and replaced with αMEM +10% FBS. Cells were harvested for experimentation 72 h after transfection. The sense (5′-3′) sequences of the siRNA oligonucleotides used are as follows: GAGGAUUGAACCAGUAUAATT (FoxO1_1), CCAGGCACCUCAUAACAAATT (FoxO1_2), CAGCAAUGAUGACUUUGAUTT (FoxO1_3), GCACCAUGAAUCUGAACGATT (FoxO3_1), CCCUCAUCUCCACACAGAATT (FoxO3_2), CGAGUUGACUGGACAUAAATT (AMPKα1), GCAACUAUCAAAGACAUACTT (AMPKα2), UUCUCCGAACGUGUCACGUTT (Scramble).

### 2.10 Statistical analyses of non-omic data

Results are means ± SEM. Statistical testing was performed using two-tailed *t*-tests when comparing two means or analysis of variance (ANOVA) with Holm Sidak test when comparing more than two means. Statistical evaluation was performed using SigmaPlot 11.0. The significance level was set at p < 0.05.

### 2.11 Additional information

Material and methods for the proteomics and metabolomics studies as well as the antibodies and primers used can be found in the Supplementary data.

## 3 Results

### 3.1 Muscle mTORC2 regulates lipid metabolism

**Figure 1:**
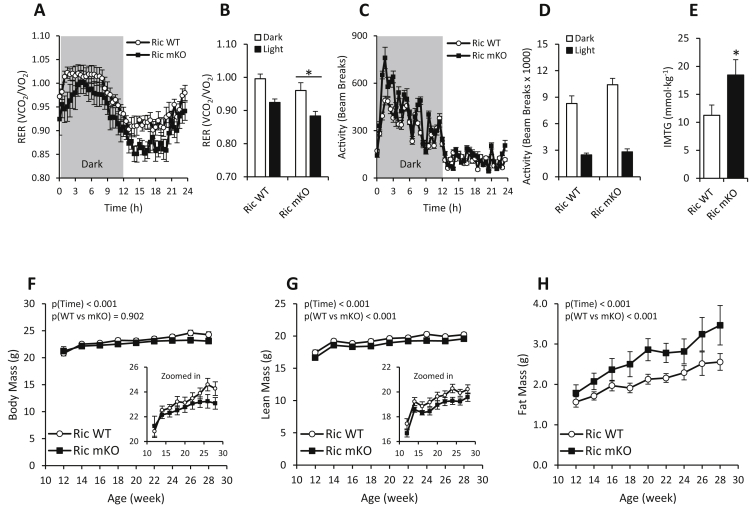
**mTORC2/Rictor in skeletal muscle regulates body composition, muscle triglyceride content and substrate utilization. (A,B)** Respiratory exchange ratio (RER) (n = 10) and **(C,D)** total activity determined over three days with 12 h light–dark cycles (n = 6). **(E)** Triglyceride content in dissected quadriceps from 12 h fasted mice (n = 6–8). **(F–H)** Body mass and fat and lean mass determined by MRI scanning at the indicated ages in ad libitum chow fed Ric WT and Ric mKO mice (n = 8–9). *p < 0.05 main effect of genotype (Ric WT vs Ric mKO). Values are means ± SEM.

Ric mKO mice had a lower respiratory exchange ratio (RER) (Figure 1A and B), indicating an increased reliance on lipid metabolism. Locomotor activity was comparable between Ric WT and Ric mKO mice (Figure 1C and D). Oxygen consumption (Ric WT: dark phase 3537 ± 132 ml h^−1^ kg^−1^; light phase 2964 ± 121 ml h^−1^ kg^−1^ vs Ric mKO: dark phase 3540 ± 151 ml h^−1^ kg^−1^; light phase 2868 ± 143 ml h^−1^ kg^−1^; n = 10) and food intake (Ric WT dark phase 2.8 ± 0.2 g; light phase 1.0 ± 0.1 g vs Ric mKO: dark phase 3.0 ± 0.2 g; light phase 1.0 ± 0.1 g; n = 6–7) were also similar between Ric WT and Ric mKO mice.

Ric mKO mice weighed the same as wildtype littermates (Figure 1F), but their lean mass was decreased (Figure 1G) and their fat mass increased (Figure 1H). At 28 weeks of age, Ric mKO mice had ∼30% (or ∼1 g) more fat mass. This was concomitant with an increase in muscle fat content of ∼65% more intramyocellular triglyceride (IMTG) in Ric mKO muscle (Figure 1E). Triglyceride (TG) concentrations in the liver were, as expected, unchanged (Figure S2A).

Metabolomic analysis of Ric WT and Ric mKO muscles identified 489 metabolites. Of those, 83 metabolites (↑55, ↓28 in Ric mKO) were significantly altered (p < 0.05) (Tables S1 and S2). Principal component analysis indicated that lack of mTORC2 activity elicits a unique biochemical profile (Figure S1B). Unbiased random forest analysis predicted the two genotypes (Ric WT vs Ric mKO) with an accuracy of 93.75% (Figure S1D) and the top 30 predictive metabolites are shown in Figure S1D. Among these, amino acids were most prominently represented (13 metabolites), followed by lipids (7 metabolites). Also, three of the five most predictive metabolites were acetylated metabolites. In general, of the significantly altered glucose metabolites the majority was decreased in Ric mKO muscle, while lipid metabolites tended to be increased (Figure S1C; Tables S1 and S2).

To understand the mechanisms behind the changes in lipid metabolites, substrate utilization and lipid storage in Ric mKO mice, we performed quantitative proteomics by tandem mass spectrometry (MS). This quantified 8,323 unique protein groups at a 1% false discovery rate (FDR) in quadriceps muscles. Of those, 30 protein groups were differentially expressed in Ric mKO muscle compared to Ric WT muscle (adjusted P < 0.05) (Tables S3). 3-ketoacyl-CoA thiolase and acyl-CoA dehydrogenase 10 (or 12), two enzymes of the β-oxidation pathway, were increased in Ric mKO muscle (Figure S1A). There was a trend (adjusted P = 0.082) for increased protein expression of ATP citrate lyase (Figure S1A), a key enzyme in synthesis of cytosolic acetyl CoA. Perilipin 3 (PLIN3), a lipid droplet (LD)-coating protein, was highly significantly (adjusted P = 0.001) regulated in Ric mKO muscles with an increased expression of 85% (Figure S1A).

### 3.2 Robust overexpression of PLIN3 in Ric mKO skeletal muscle

**Figure 2:**
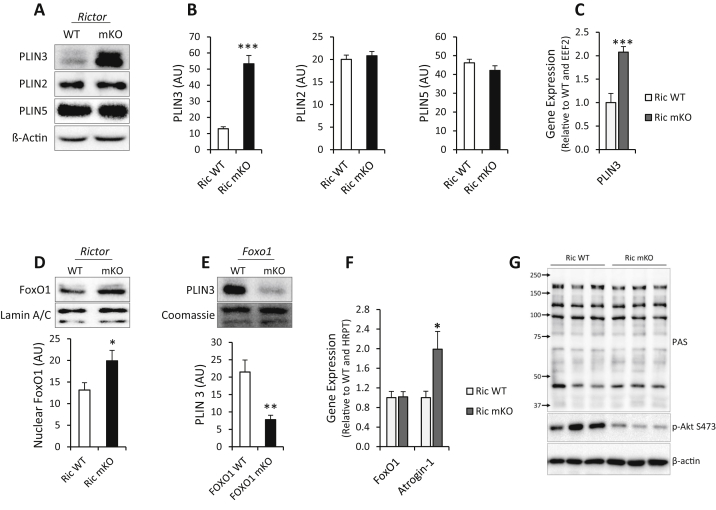
**mTORC2/Rictor regulates Perilipin 3 via FoxO1 in skeletal muscle. (A,B)** Representative western blots and quantitative analysis of PLIN3, PLIN2 and PLIN5 in Ric WT and Ric mKO quadriceps muscle (n = 17–18). **(C)** Gene expression of PLIN3 in Ric WT and Ric mKO quadriceps (n = 6–7). **(D)** Representative western blot and quantitative analysis of nuclear FoxO1 enrichment in Ric WT and Ric mKO quadriceps (n = 12). **(E)** Gene expression of FoxO1 and Atrogin-1 in Ric WT and Ric mKO quadriceps (n = 7–9). **(F)** Representative western blot and quantitative analysis of PLIN3 in muscle-specific FoxO1 WT and FoxO1 KO muscles (n = 6). **(G)** Western blots with indicated antibodies in quadriceps lysates from fasted-refed Ric WT and Ric mKO mice. ***p < 0.001, **p < 0.01, *p < 0.05 are genotype differences (Ric WT vs Ric mKO) as indicated. Values are means ± SEM.

Loss-of-function studies have implicated PLIN3 as a regulator of lipid storage [6], [7], [39]. Given the increase in IMTG levels in muscle of Ric mKO muscle, we focused on PLIN3. Protein expression of PLIN3, measured by immunoblotting, was ∼5-fold higher in Ric mKO muscle, confirming the proteome data (Figure 2A and B). Gene expression of PLIN3 was also elevated (∼2-fold) in Ric mKO muscle (Figure 2C). The protein expression of the perilipin family members, PLIN2 and PLIN5, which have been implicated in the regulation of muscle lipid storage [5], [15], [28] was not altered in Ric mKO muscle (Figure 2A and B).

### 3.3 FoxO1 is necessary for PLIN3 expression in skeletal muscle

In the liver, mTORC2 signals to the transcription factor forkhead box protein O1 (FoxO1) via Akt [14]. We hypothesized that in muscle PLIN3 expression is FoxO1-dependent. Ric mKO muscle had increased nuclear FoxO1 content (Figure 2D) and gene expression of the FoxO1 target, atrogin-1, was increased (Figure 2F), indicating increased transcriptional activity of FoxO1. In addition, protein expression of PLIN3 was reduced by 64% in muscle from muscle-specific FoxO1 knockout mice (Figure 2E). Akt activity judged with the PAS antibody was similar in Ric WT and Ric mKO muscle from fasted-refed mice (Figure 2G), which was consistent with comparable phosphorylation levels of the Akt target site S256 on FoxO1 in Ric WT and Ric mKO muscle (Figure S2B).

### 3.4 AMPK activity is increased in Ric mKO mice and AMPK is necessary for normal PLIN3 expression and IMTG levels

**Figure 3:**
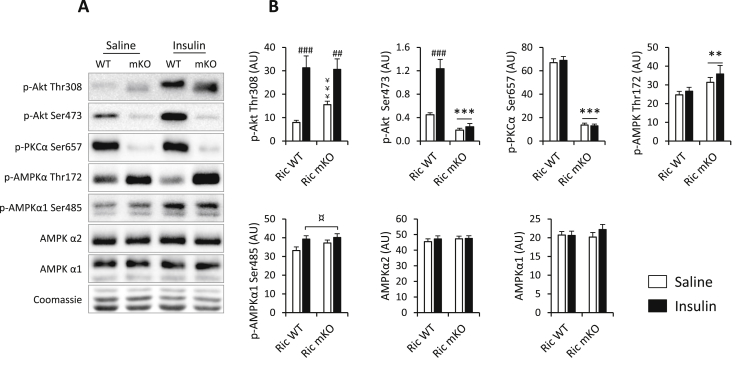
**AMPK activity is increased in Ric mKO muscle. (A,B)** Representative blots and quantitative analysis of indicated phosphorylation sites and total proteins in quadriceps muscle from Ric WT and Ric mKO mice 10 min after i.p. saline or insulin (2.5 U/kg) injection (n = 11–12, except for Akt2 blots where the n = 8–9). ***p < 0.001, **p < 0.01, and *p < 0.05 main effects of genotype (Ric WT vs Ric mKO); ###p < 0.001, ##p < 0.01 are insulin effects compared to corresponding basal; ¤p < 0.05 main effect of insulin; ￥￥￥p < 0.001 genotype effect within saline injection. Values are means ± SEM.

AMPK has been shown to modulate FoxO1 transcriptional activity [44]. In addition, our metabolomic data revealed an increase in endogenous AICAR, an AMPK activator, in Ric mKO muscle, along with alterations in a number of other purine metabolites (Tables S1). Others have recently reported increased AMPK activity following RICTOR knockdown in smooth muscle cells [13]. We assessed AMPK activity by measuring AMPKα Thr172 phosphorylation [38] in muscle from saline or insulin stimulated Ric WT and Ric mKO mice. Insulin increased p-Akt Thr308 (Figure 3A and B). Decreased p-PKCα Ser657 and p-Akt Ser473 in Ric mKO muscle is consistent with RICTOR deletion (Figure 3A and B). AMPKα Thr172 phosphorylation was elevated in Ric mKO mice by ∼30% with both saline and insulin stimulation compared to Ric WT mice (Figure 3A and B), while protein expression of either the AMPKα1 or AMPKα2 subunits were similar (Figure 3A and B).

Akt can inhibit AMPK activation by phosphorylating AMPK on the Ser485 residue [16], but while insulin injection increased AMPK Ser485 phosphorylation (main effect; Figure 3A and B), there was no difference in phosphorylation of AMPK Ser485 between Ric WT and Ric mKO muscles (Figure 3A and B).

**Figure 4:**
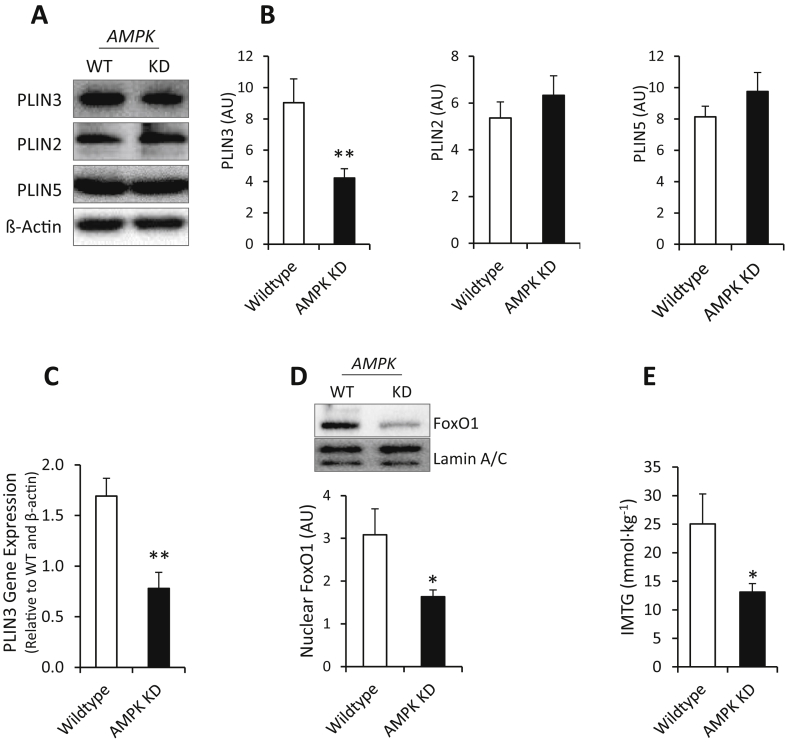
**AMPK is necessary for PLIN3 expression. (A,B)** Representative western blots and quantitative analysis of PLIN3, PLIN2 and PLIN5 protein expression from WT quadriceps muscle or muscle over-expressing a kinase-dead AMPKα_2_ construct (AMPK KD) (n = 11). **(C)** PLIN3 mRNA levels in wildtype and AMPK KD quadriceps muscle determined by qPCR (n = 11). **(D)** Representative western blot and quantitative analysis of nuclear FoxO1 protein content in AMPK wildtype and KD quadriceps (n = 9–11). **(E)** Triglyceride concentration in dissected quadriceps from fed AMPK wildtype and KD mice (n = 11). **p < 0.01, *p < 0.05 effects of genotype (Wildtype vs AMPK KD). Values are means ± SEM.

In muscles over-expressing a kinase-dead α2 AMPK subunit (AMPK KD), which reduces overall AMPK activity [19], [30], PLIN3 protein expression was decreased to ∼47% of the WT level (Figure 4A and B). This was accompanied by a ∼2-fold reduction in PLIN3 gene expression (Figure 4C) and a decrease in nuclear FoxO1 content (Figure 4D) in AMPK KD muscles. Akin to Ric mKO muscle, changes in PLIN3 expression were independent of changes in PLIN2 and PLIN5 protein content in AMPK KD muscles (Figure 4A and B). AMPK KD muscle exhibited a ∼50% decrease in IMTG levels (Figure 4E). In summary, the data from the AMPK KD muscle were reciprocal to the findings in Ric mKO muscle.

### 3.5 AMPK agonism induces PLIN3 expression

**Figure 5:**
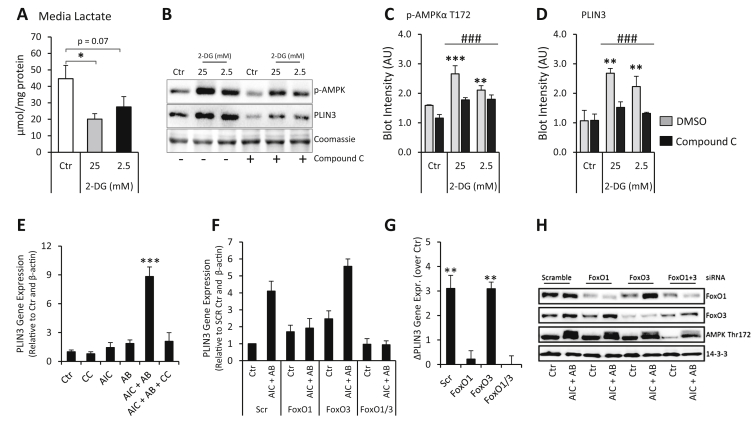
**AMPK is sufficient for PLIN3 expression in muscle cells. (A)** L6 myoblasts were incubated with 25 mM and 2.5 mM 2-deoxyglucose (2-DG) for 16 h and media lactate was determined (n = 4). **(B–D)** Representative western blots and quantitative analysis of p-AMPKα T172 and PLIN3 in L6 myoblasts treated as described in (A) ± Compound C (1 μM) (n = 3). **(E)** PLIN3 mRNA levels in L6 myotubes following 16 h of no treatment (Ctr), treatment with 2 mM AICAR (AIC), 100 μM A-769662 (AB) or both compounds (AIC + AB). Where indicated L6 cells were also treated with 10 μM Compound C (CC) (n = 4–6). **(F,G)** PLIN3 expression in L6 myoblasts after Ctr or AIC + AB treatment ± siRNA-mediated knockdown of FoxO1, FoxO3 or both isoforms (n = 4–6), along with **(H)** representative western blots with the indicated antibodies. ***p < 0.001, **p < 0.01, *p < 0.05 different from control (Ctr) or scramble (Scr); ###p < 0.001 main effect of Compound C. Values are means ± SEM.

We next asked whether AMPK activation is sufficient to increase PLIN3 protein expression in muscle cells. We treated muscle cells with the glycolysis inhibitor 2-deoxyglucose (2-DG) to activate the energy sensor AMPK. 2-DG treatment decreased glycolysis, as assessed by media lactate concentration (Figure 5A) and induced AMPK phosphorylation (Figure 5B and C). This increase in AMPK activity was associated with an increase in PLIN3 protein expression (Figure 5B and D). The AMPK inhibitor Compound C inhibited the 2-DG-induced increase in AMPK phosphorylation as well as PLIN3 expression (Figure 5B–D). AMPK activation with combined treatment with AMPK agonists, AICAR and A769662, induced a marked increase in PLIN3 gene expression (Figure 5E) in muscle cells. This AMPK-induced PLIN3 expression was blunted by the AMPK inhibitor Compound C (Figure 5E) and by siRNA mediated knockdown of AMPKα subunits (Figure S3A). We further hypothesized that AMPK-mediated PLIN3 expression requires FoxO1. AMPK agonism in conjunction with siRNA-mediated knockdown of FoxO1 and FoxO3 revealed that FoxO1, but not FoxO3, is obligatory for AMPK-mediated PLIN3 expression in muscle cells (Figure 5F–H and Figure S3B and C).

### 3.6 PLIN3 overexpression increases muscle cell triglycerides

**Figure 6:**
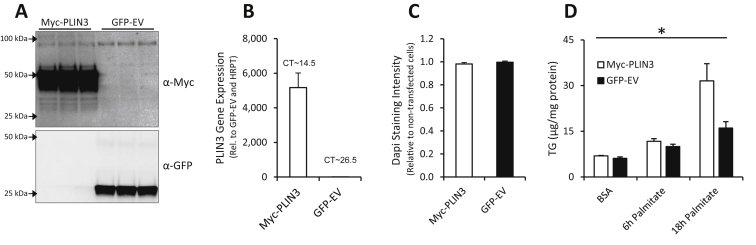
**PLIN3 is sufficient to increase triglyceride content in muscle cells. (A)** Immunoblots probing for myc-tag and GFP (n = 3) and **(B)** gene expression of PLIN3 (n = 4) in C2C12 myotubes 24 h after transfection with indicated constructs. **(C)** Dapi staining intensity relative to untreated myotubes in 24 h transfected C2C12 myotubes (n = 7). **(D)** Triglyceride (TG) levels in transfected myotubes treated with 2% BSA only or with 750 μM palmitate as indicated (n = 3). *p < 0.05 main effects of Myc-PLIN3 vs GFP-EV. Values are means ± SEM.

To understand whether the increase in IMTG levels in RICTOR-deficient muscles is directly linked to PLIN3, we overexpressed PLIN3 (Myc-PLIN3) and a control GFP-tagged empty vector (GFP-EV) constructs in muscle cells (Figure 6A and B). Transfection with Myc-PLIN3 or GFP-EV had no apparent effect on cell viability, as judged with Dapi staining (Figure 6C). Incorporation of palmitate into TG increased 2-fold over control GFP-EV after 16 h in PLIN3 overexpressing cells (Figure 6D). This indicates that PLIN3 is sufficient to increase TG content and therefore suggests that in Ric mKO muscle the increase in PLIN3 drives the increase in IMTG.

## 4 Discussion

We here provided new evidence that mTORC2 regulates substrate utilization, lipid metabolites and lipid storage, emphasizing the importance of mTORC2 in muscle metabolism. With regards to lipid storage, we identified a hitherto unknown link between PLIN3 and mTORC2 activity, as mTORC2, via PLIN3, is a negative regulator of IMTG levels. In contrast, AMPK appears to be a positive regulator of PLIN3 expression and IMTG content.

Previously it has been shown that PLIN3 deletion decreased TG content [6], [7], [39] and inhibited the maturation of lipid droplets [7] in cells. Virus-mediated knockdown of PLIN3 in the liver of high-fat diet fed mice improved hepatic steatosis along with glucose homeostasis [9]. Our present results complement these findings, as we show that PLIN3 is sufficient to increase TG content. Moreover, it was previously unknown what regulates PLIN3 expression in muscle. PLIN2 and PLIN5 are regulated by PPARδ and HFD in muscle, while PLIN3 was unresponsive [4]. Our data indicates that PLIN3 expression requires, at least partially, FoxO1 and that AMPK is a positive regulator of PLIN3. Whether the PLIN3-mediated increase in IMTG in Ric mKO muscle contributes to the insulin resistance in Ric mKO mice [22] will have to be determined.

In the current study we focused on PLIN3, but other RICTOR-regulated proteome “hits” are of future interest. The protein phosphatase 1 regulatory subunit 1A (Ppp1r1a) was increased ∼1.6-fold in Ric mKO muscle. Ppp1r1a may play a regulatory role in glycogen metabolism [32] and Ric mKO mice have elevated glycogen levels [22]. Another observation was the marked decrease in paraoxonase 3 (PON3) protein content in Ric mKO muscle. PON3 is a member of the paraoxonase family, which includes PON1 and PON2. PON proteins are generally known as high-density lipoprotein (HDL)–associated proteins that have anti-oxidative, cardioprotective and antiatherogenic properties [8]. In addition, PON proteins have been linked to insulin sensitivity [18], [41]. This is noteworthy given the previously reported insulin resistance in the Ric mKO mice [22]. Deficiency of PON1 leads to glucose intolerance as well as glycogen accumulation in muscle, while forced overexpression of PON1 in muscle cells enhances GLUT4 expression [23].

Similarly, the metabolomics data should provide inspiration for future follow-up studies. With regards to glycogen metabolism, it is of note that multiple glycogen metabolites including maltotetraose, maltotriose, and maltose were diminished in Ric mKO muscle. Maltotriose and maltose were among the 30 most predictive and their low levels may reflect limited glycogen availability or diminished breakdown. Also among the top 30 list were oxidized glutathione (GSSG) and cysteine-glutathione disulfide, both elevated in Ric mKO muscle. This could indicate increased free radical exposure. In agreement, the oxidative stress marker ophthalmate (a tripeptide analogue of glutathione in which cysteine is replaced by 2-aminobutyrate) also accumulated in Ric mKO muscles. Conversely, histidine-derived antioxidants, such as carnosine, N-acetylcarnosine, and anserine were significantly diminished in Ric mKO muscle compared to Ric WT counterparts. These metabolic “hits” will need to be validated, but together with other lines of evidence they may give rise to future hypotheses.

FoxO1 is transcriptionally inactive in the cytosol and active in the nucleus. FoxO1 localization is tightly regulated and matched to the cell's energy status by posttranslational modifications, such as phosphorylation. Akt-mediated phosphorylation of FoxO1 leads to nuclear export of FoxO1 [1]. Conversely, diminished Akt activity, with the subsequent decrease in FoxO1 phosphorylation, results in an enrichment of FoxO1 in the nucleus. As in the RICTOR-deficient liver [14], we detected increased nuclear content of FoxO1 in Ric mKO muscle. Yet, our data point to tissue differences (muscle vs liver) with regards to how RICTOR KO impacts FoxO1. While Akt-mediated FoxO1 phosphorylation was impaired in the liver [14], [43], we could not detect decreased phosphorylation in muscle, consistent with a previous report [3]. Akt activity judged with a pan-Akt substrate antibody was normal in Ric mKO muscle, as was the Akt target site on AMPK. Previously, we have shown that phosphorylation of the Akt substrates, AS160 and GSK3, was not impaired in Ric mKO muscle [22]. It cannot be ruled out that Akt activity towards specific substrates is blunted, but it seems that *in vivo* Akt activity is largely normal in Ric mKO muscle.

AMPK regulation of FoxO1 could present an alternative. Yun et al. have proposed that AMPK phosphorylates FoxO1 to increase FoxO1 transcriptional activity [44]. What causes the increase in AMPK activity observed by us presently in muscle and by others previously in cells [13] requires future investigations. It is not clear whether the 50% increase in endogenous AICAR is sufficient to affect AMPK activity in Ric mKO muscle and we cannot rule out that other upstream regulators, changes in the overall energy state, or stress signaling pathways are underpinning the elevation in AMPK activity in Ric mKO muscle.

Our finding that AMPK is involved in muscle lipid storage is a bit perplexing, given that AMPK activity is generally thought to promote glucose and lipid catabolism to provide energy substrates in times of cellular energy paucity. Activation of AMPK increases both glucose and lipid oxidation in resting muscle [29], but whether AMPK also plays a role in energy storage has not been thoroughly investigated. However, chronic activation of AMPK gamma1 in muscle increases glycogen levels [2], gain-of function gamma2 mutation in the heart causes massive glycogen accumulation [27] and AICAR-induced AMPK activation has also been shown to increase muscle glycogen in mice [17]. The role of AMPK in lipid storage IMTG is unclear, but our data should spur future research into AMPK-regulated IMTG turnover, especially since exercise training (i.e., repeated AMPK activation) can lead to elevated IMTG levels [40].

## 5 Conclusions

In conclusion, we have characterized new aspects of mTORC2 and AMPK biology in muscle lipid metabolism by showing that mTORC2 and AMPK are, respectively, negative and positive regulators of lipid storage via PLIN3. In addition, lack of mTORC2 activity in muscle caused a greater reliance on fat as an energy substrate and re-partitioning of lean to fat mass.

## Author contributions

MK and EAR conceived and designed the studies and wrote the manuscript. MK performed all the RICTOR mKO mouse studies, some cell experiments and the tissue and plasma analyses, along with data analyses. BJP performed the proteomics and together with RC the subsequent data processing and statistical analysis. AS performed most of the AMPK KD studies. JRK, DJF and KCT performed the RT-qPCR in the RICTOR mKO muscles. LSH assisted in the RICTOR mKO studies. AMF carried out some of the AMPK KD studies. NJH performed the AMPK knockdown studies in cells and helped with the proteomics. PS performed the genotyping of the RICTOR mKO and AMPK KD mice. JJ, BK, DEJ and EAR supervised and performed part of the studies and provided critical guidance in the manuscript preparation. MAR generated the RICTOR mKO mice. All authors read and provided comments on the final version of the manuscript.
