# Supplementary material for: mTORC2 and AMPK differentially regulate muscle triglyceride content via Perilipin 3
Source: Mol Metab. 2016 Jun 22;5(8):646–55. doi: 10.1016/j.molmet.2016.06.007 (PMC5021677; doi:10.1016/j.molmet.2016.06.007)
Supplement: Supplementary file 3 [file mmc3.pdf]

Figure S1

A

Protein Description	Gene Symbol	Expression in Ric mKO	Adjusted *P*-value
3-ketoacyl-CoA thiolase, mitochondrial	Acaa2	+23%	0.035
Acyl-CoA dehydrogenase family member 10/12	Acad10/12	+24%	0.035
Perilipin-3	Plin3	+85%	0.001
ATP-citrate synthase	Acly	+30%	0.082

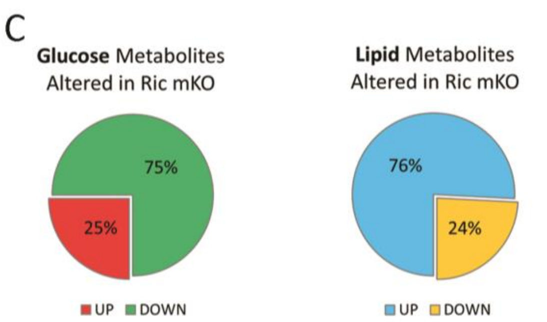

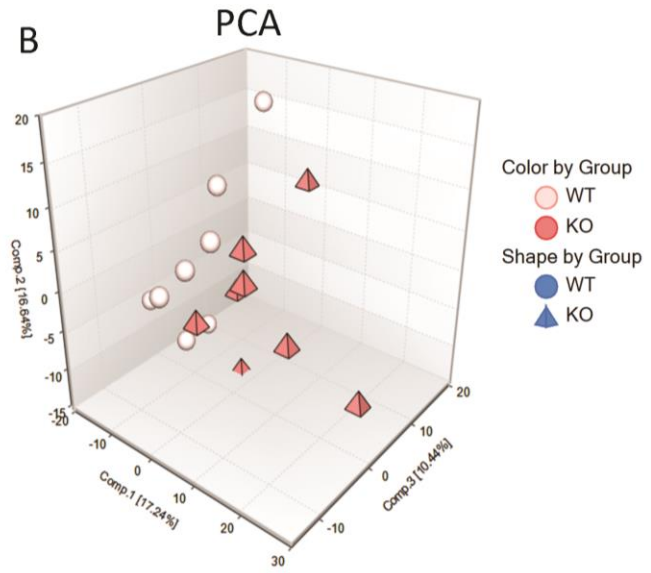

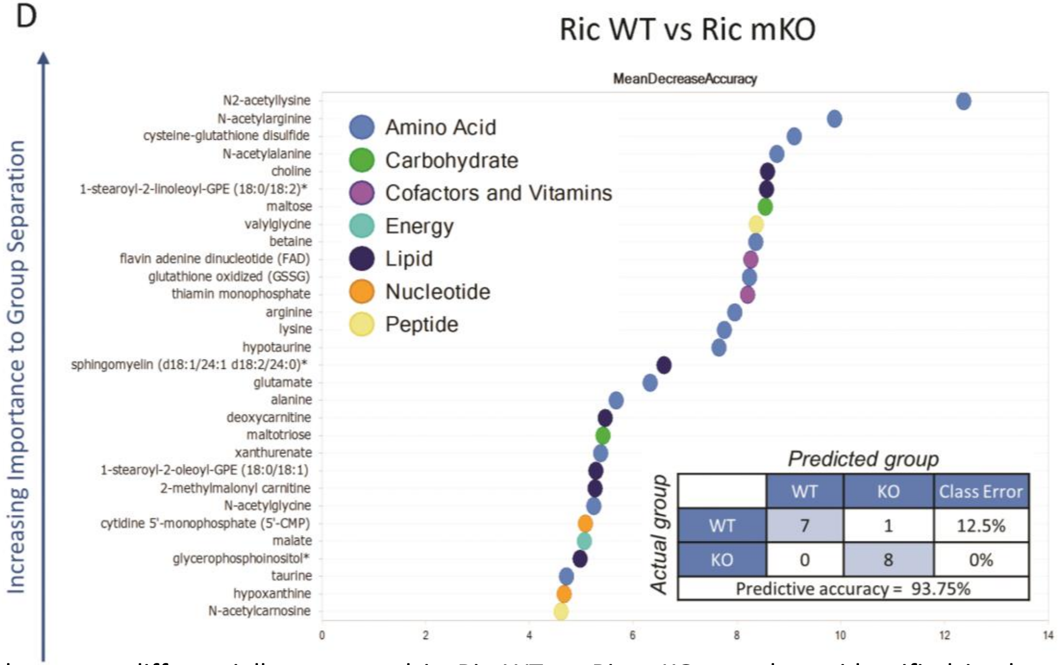

Figure S1 related to Figure 1: (A) Lipid metabolism proteins that were differentially expressed in Ric WT vs Ric mKO muscle as identified in the proteome analysis by MS. **(B)** Principal component analysis of the metabolome revealed a distinct separation between Ric WT and Ric mKO muscles suggesting that mTORC2-deficient muscle possess a unique biochemical signature. **(C)** Directional changes of glucose and lipid metabolites that were altered based on p<0.1 as identified by the metabolomic screen. **(D)** Random forest analysis (a supervised classification technique based on an ensemble of decision trees) resulted in a predictive accuracy of 93.75% (shown in the inset table). A value of 93.75% is greater than one would expect by random chance alone (50%), suggesting that these metabolites may be of interest as candidate biomarkers associated with mTORC2 expression. Listed are the top 30 metabolites based on their importance in separating the two groups.

Figure S2

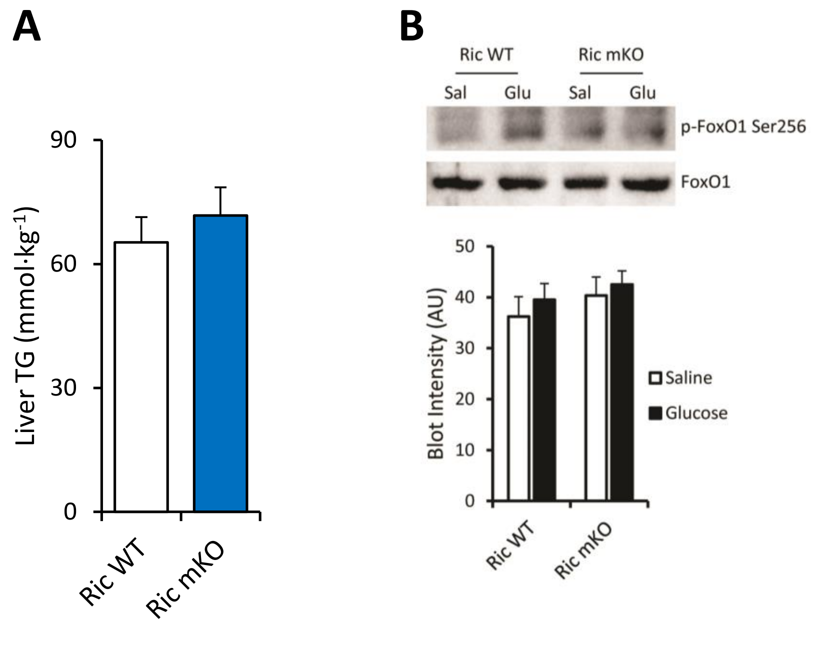

Figure S2 related to Figure 1 and 2: (A) Liver triglyceride levels (n = 6-8). **(B)** Representative blot and quantification of p-FoxO1 Ser256 in quadriceps muscle lysate from Ric WT and Ric mKO mice that had been injected with saline or glucose (n = 8-9). FoxO1 total serves as loading control.

Figure S3

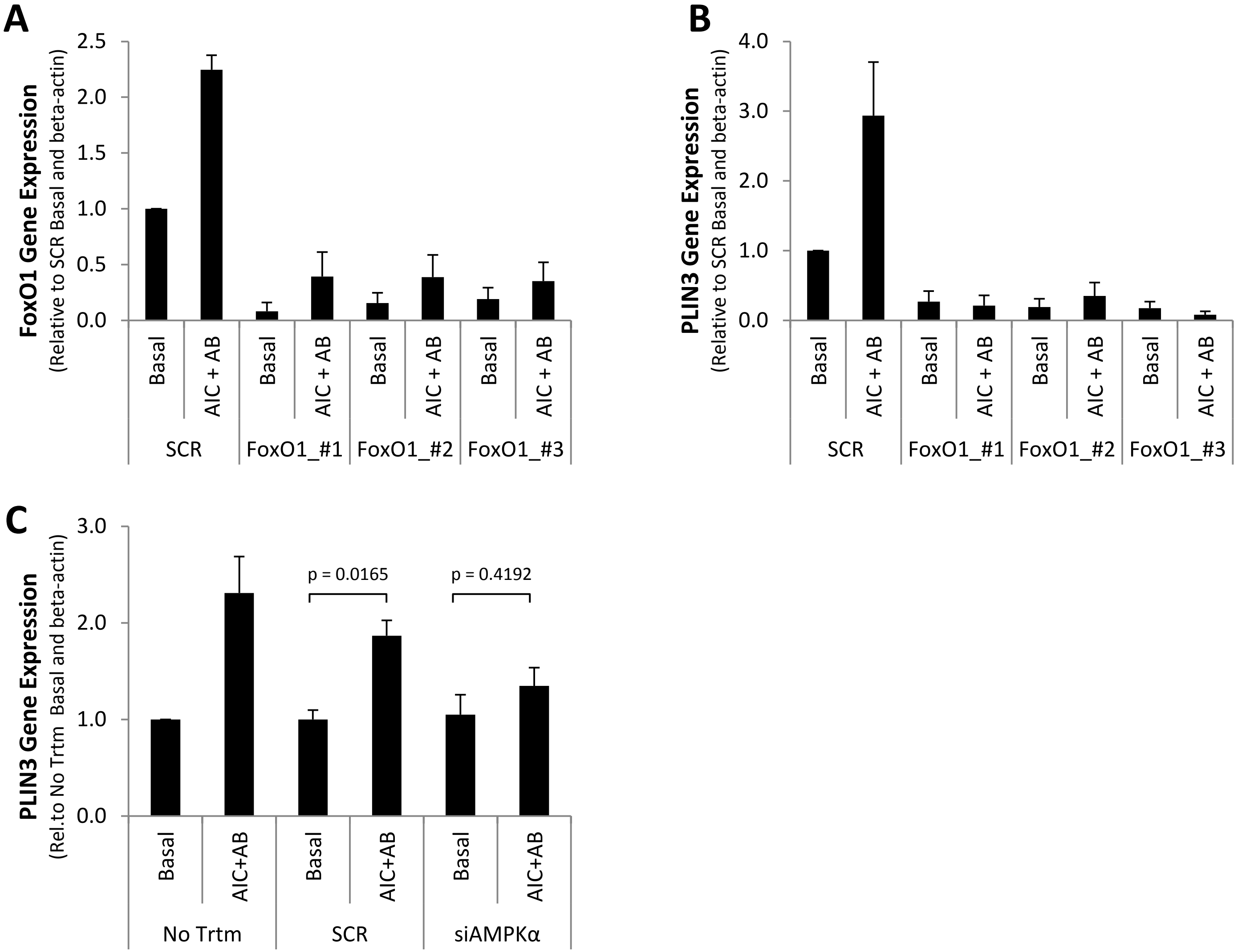

Figure S3 related to Figure 5: (A,B) The effect of FoxO1 knockdown using individual FoxO1 (FoxO1 (1), FoxO1 (2), FoxO1 (3)) siRNA sequences (methods online) on gene expression of FoxO1 and PLIN3 under control (Ctr) conditions and in response to AMPK activation by AICAR (AIC) plus A769662 (AB) (n = 3) as described in methods online. **(C)** The effect of AMPKα knockdown on PLIN3 expression following AMPK activation by AICAR (AIC) plus A769662 (AB).

Table S1

ACC#	Protein Description	Gene Symbol	Ric mKO / Ric WT
Q62087	Serum paraoxonase/lactonase 3	Pon3	-101%
Q9JME5	AP-3 complex subunit beta-2	Ap3b2	-63%
Q9Z0J4	Nitric oxide synthase	Nos1	-50%
Q9Z0J4-2	N-NOS-2 Nitric oxide synthase		
Q9Z0J4-3	NNOS beta Nitric oxide synthase		
Q9Z0J4-4	NNOS gamma Nitric oxide synthase		
Q9Z0J4-5	NNOS mu Nitric oxide synthase		
O08689	Growth/differentiation factor 8	Mstn	-47%
P55012	Solute carrier family 12 member 2	Slc12a2	-31%
Q8R1G2	Carboxymethylenebutenolidase homolog	Cmbl	-29%
P82350	Alpha-sarcoglycan	Sgca	-29%
Q9QUH0	Glutaredoxin-1	Glrx	-28%
D3Z7P3	Glutaminase kidney Iso	Gls	-25%
D3Z7P3-2	Iso 2 Glutaminase kidney Iso		
P29758	Ornithine aminotransferase	Oat	-22%
Q9DAV9	Trimeric intracellular cation channel type B	Tmem38b	-22%
Q91YW3	DnaJ homolog subfamily C member 3	Dnajc3	-22%
Q05BC3	Echinoderm microtubule-associated protein-like 1	Eml1	-19%
Q05BC3-2	Iso 2 Echinoderm microtubule-associated protein-like 1		
Q05BC3-3	Iso 3 Echinoderm microtubule-associated protein-like 1		
Q9DB05	Alpha-soluble NSF attachment protein	Napa	18%
Q6VGS5	Protein Daple	Ccdc88c	19%
Q6VGS5-2	Iso 2 Protein Daple		
Q9JJZ3	SH3 and multiple ankyrin repeat domains protein 3	Shank3	
Q8R164	Valacyclovir hydrolase	Bphl	22%
Q61362	Chitinase-3-like protein 1	Chi3l1	23%
Q8BWT1	3-ketoacyl-CoA thiolase mitochondrial	Acaa2	
Q8K370	Acyl-CoA dehydrogenase family member 10	Acad10	24%
D3Z2B3	Acyl-CoA dehydrogenase family member 12	Acad12	
P15105	Glutamine synthetase	Glul	26%
Q5NBX1	Protein cordon-bleu	Cobl	37%
Q5NBX1-2	Iso 2 Protein cordon-bleu		
Q5NBX1-3	Iso 3 Protein cordon-bleu		
Q5NBX1-4	Iso 4 Protein cordon-bleu		
Q3KNY0	Ig-like and fibronectin type III domain-containing protein 1	Igfn1	37%
Q3KNY0-2	Iso 2 Ig-like and fibronectin type III domain-containing protein 1		
Q3KNY0-3	Iso 3 Ig-like and fibronectin type III domain-containing protein 1		
Q3KNY0-4	Iso 4 Ig-like and fibronectin type III domain-containing protein 1		
Q9QXY6	EH domain-containing protein 3	Ehd3	45%
Q9EQP2	EH domain-containing protein 4	Ehd4	
Q3KNY0	Ig-like and fibronectin type III domain-containing protein 1	Igfn1	49%
P17563	Selenium-binding protein 1	Selenbp1	53%
P16015	Carbonic anhydrase 3	Ca3	62%
Q9ERT9	Protein phosphatase 1 regulatory subunit 1A	Ppp1r1a	65%
P09541	Myosin light chain 4	Myl4	72%
Q9DBG5	Perilipin-3	Plin3	85%

Table S1: Protein groups differentially expressed in Ric mKO muscle compared to Ric WT muscle (adjusted P value < 0.05) (n = 8). Gray bars on the left encompass the protein groups that shared peptide sequences identified and quantified by unbiased MS-based proteomics.

Table S2

Biochemical Name	KEGG	HMDB	PubChem	Ric mKO / Ric WT	Super Pathway	Sub Pathway
ergothioneine	C05570	HMDB03045	3032311	10.45	Xenobiotics	Food Component/Plant
galactose 1-phosphate	C00446	HMDB00645	123912	2.66	Carbohydrate	Fructose, Mannose and Galactose Metabolism
S-lactoylglutathione	C03451	HMDB01066	440018	2.47	Amino Acid	Glutathione Metabolism
riboflavin (Vitamin B2)	C00255	HMDB00244	493570	2.02	Cofactors and Vitamins	Riboflavin Metabolism
phosphopantetheine	C01134	HMDB01416	987	1.98	Cofactors and Vitamins	Pantothenate and CoA Metabolism
xanthosine	C01762	HMDB00299	64959	1.88	Nucleotide	Purine Metabolism, (Hypo)Xanthine/Inosine containing
cysteine-glutathione disulfide	--	HMDB00656	4247235	1.84	Amino Acid	Glutathione Metabolism
thiamin diphosphate	C00068	HMDB01372	1132	1.75	Cofactors and Vitamins	Thiamine Metabolism
1-stearoyl-2-oleoyl-GPE (18:0/18:1)	--	--	--	1.69	Lipid	Phospholipid Metabolism
gamma-glutamylglutamate	C05282	HMDB11737	92865	1.66	Peptide	Gamma-glutamyl Amino Acid
leucylglycine	--	--	79070	1.62	Peptide	Dipeptide
acetyl CoA	C00024	HMDB01206	444493	1.55	Lipid	Fatty Acid Metabolism
hypotaurine	C00519	HMDB00965	107812	1.54	Amino Acid	Methionine, Cysteine, SAM and Taurine Metabolism
choline	C00114	HMDB00097	305	1.53	Lipid	Phospholipid Metabolism
valylglycine	--	--	136487	1.52	Peptide	Dipeptide
1-palmitoyl-GPI (16:0)*	--	HMDB61695	--	1.49	Lipid	Lysolipid
AICA ribonucleotide	C04677	HMDB01517	65110	1.48	Nucleotide	Purine Metabolism, (Hypo)Xanthine/Inosine containing
1-docosahexaenoylglycerol (22:6)	--	HMDB11587	--	1.44	Lipid	Monoacylglycerol
N-acetylglycine	--	HMDB00532	10972	1.42	Amino Acid	Glycine, Serine and Threonine Metabolism
1-palmitoyl-2-oleoyl-GPE (16:0/18:1)	--	HMDB05320	5283496	1.42	Lipid	Phospholipid Metabolism
1-palmitoyl-2-linoleoyl-GPE (16:0/18:2)	--	HMDB05322	9546747	1.42	Lipid	Phospholipid Metabolism
1-stearoyl-2-linoleoyl-GPE (18:0/18:2)*	--	--	9546749	1.41	Lipid	Phospholipid Metabolism
spermidine	C00315	HMDB01257	1102	1.40	Amino Acid	Polyamine Metabolism
gamma-glutamylphenylalanine	--	HMDB00594	111299	1.38	Peptide	Gamma-glutamyl Amino Acid
2-methylmalonyl carnitine	--	HMDB13133	53481628	1.38	Lipid	Fatty Acid Synthesis
hydroxybutyrylcarnitine*	--	HMDB13127	53481617	1.38	Lipid	Fatty Acid Metabolism(Acyl Carnitine)
glycerophosphoinositol*	--	--	--	1.38	Lipid	Phospholipid Metabolism
squalene	C00751	HMDB00256	638072	1.35	Lipid	Sterol
ethanolamine	C00189	HMDB00149	700	1.32	Lipid	Phospholipid Metabolism
adenosine 3',5'-diphosphate	C00054	HMDB00061	159296	1.32	Nucleotide	Purine Metabolism, Adenine containing
N-acetylalanine	C02847	HMDB00766	88064	1.30	Amino Acid	Alanine and Aspartate Metabolism
thiamin monophosphate	C01081	HMDB02666	3382778	1.30	Cofactors and Vitamins	Thiamine Metabolism
glutathione, oxidized (GSSG)	C00127	HMDB03337	65359	1.29	Amino Acid	Glutathione Metabolism
suberate (octanedioate)	C08278	HMDB00893	10457	1.29	Lipid	Fatty Acid, Dicarboxylate
flavin adenine dinucleotide (FAD)	C00016	HMDB01248	643975	1.28	Cofactors and Vitamins	Riboflavin Metabolism
xanthine	C00385	HMDB00292	1188	1.25	Nucleotide	Purine Metabolism, (Hypo)Xanthine/Inosine containing
flavin mononucleotide (FMN)	C00061	HMDB01520	710	1.25	Cofactors and Vitamins	Riboflavin Metabolism
hypoxanthine	C00262	HMDB00157	790	1.24	Nucleotide	Purine Metabolism, (Hypo)Xanthine/Inosine containing
pyridoxal phosphate	C00018	HMDB01491	1051	1.24	Cofactors and Vitamins	Vitamin B6 Metabolism
glutamate	C00025	HMDB00148	611	1.23	Amino Acid	Glutamate Metabolism
arachidonate (20:4n6)	C00219	HMDB01043	444899	1.23	Lipid	Polyunsaturated Fatty Acid (n3 and n6)
betaine	C00719	HMDB00043	247	1.22	Amino Acid	Glycine, Serine and Threonine Metabolism
1-palmitoyl-2-linoleoyl-GPC (16:0/18:2)*	--	--	5287971	1.21	Lipid	Phospholipid Metabolism
malate	C00149	HMDB00156	525	1.20	Energy	TCA Cycle
cytidine 5'-monophosphate (5'-CMP)	C00055	HMDB00095	6131	1.17	Nucleotide	Pyrimidine Metabolism, Cytidine containing
1-stearoyl-2-oleoyl-GPS (18:0/18:1)	--	--	9547087	1.15	Lipid	Phosphatidylserine (PS)
sphingomyelin (d18:1/24:1, d18:2/24:0)*	--	--	--	1.15	Lipid	Sphingolipid Metabolism
arabonate/xylonate	--	--	--	1.14	Carbohydrate	Pentose Phosphate Pathway
1-stearoyl-2-linoleoyl-GPC (18:0/18:2)*	--	--	--	1.14	Lipid	Phospholipid Metabolism
guanosine 5'- monophosphate (5'-GMP)	C00144	HMDB01397	6804	1.14	Nucleotide	Purine Metabolism, Guanine containing
kynurenine	C00328	HMDB00684	161166	1.11	Amino Acid	Tryptophan Metabolism
creatinine	C00791	HMDB00562	588	1.11	Amino Acid	Creatine Metabolism
phosphate	C00009	HMDB01429	1061	1.07	Energy	Oxidative Phosphorylation
creatine	C00300	HMDB00064	586	1.05	Amino Acid	Creatine Metabolism
taurine	C00245	HMDB00251	1123	1.03	Amino Acid	Methionine, Cysteine, SAM and Taurine Metabolism

Table S2: Metabolites that were increased ($p < 0.05$) in Ric mKO compared to Ric WT muscle ($n = 8$).

Table S3

Biochemical Name	KEGG	HMDB	PubChem	Ric mKO / Ric WT	Super Pathway	Sub Pathway
1,2-dipalmitoyl-GPC (16:0/16:0)	--	HMDB00564	452110	**0.93**	Lipid	Phospholipid Metabolism
pipecolate	C00408	HMDB00070	849	**0.92**	Amino Acid	Lysine Metabolism
alanine	C00041	HMDB00161	5950	**0.90**	Amino Acid	Alanine and Aspartate Metabolism
anserine	C01262	HMDB00194	112072	**0.89**	Peptide	Dipeptide Derivative
N-acetylneuraminate	C00270	HMDB00230	439197	**0.87**	Carbohydrate	Aminosugar Metabolism
methionine sulfoxide	C02989	HMDB02005	158980	**0.86**	Amino Acid	Methionine, Cysteine, SAM and Taurine Metabolism
carnosine	C00386	HMDB00033	439224	**0.85**	Peptide	Dipeptide Derivative
aspartate	C00049	HMDB00191	5960	**0.84**	Amino Acid	Alanine and Aspartate Metabolism
N-methylproline	--	--	557	**0.83**	Amino Acid	Urea cycle; Arginine and Proline Metabolism
4-guanidinobutanoate	C01035	HMDB03464	500	**0.80**	Amino Acid	Guanidino and Acetamido Metabolism
deoxycarnitine	C01181	HMDB01161	134	**0.80**	Lipid	Carnitine Metabolism
N6,N6,N6-trimethyllysine	C03793	HMDB01325	440120	**0.77**	Amino Acid	Lysine Metabolism
arginine	C00062	HMDB00517	232	**0.76**	Amino Acid	Urea cycle; Arginine and Proline Metabolism
galactitol (dulcitol)	C01697	HMDB00107	11850	**0.76**	Carbohydrate	Fructose, Mannose and Galactose Metabolism
betaine aldehyde	C00576	HMDB01252	249	**0.75**	Amino Acid	Glycine, Serine and Threonine Metabolism
maltotetraose	C02052	HMDB01296	446495	**0.74**	Carbohydrate	Glycogen Metabolism
lysine	C00047	HMDB00182	5962	**0.73**	Amino Acid	Lysine Metabolism
xanthurenate	C02470	HMDB00881	5699	**0.70**	Amino Acid	Tryptophan Metabolism
N-acetylcarnosine	--	HMDB12881	9903482	**0.70**	Peptide	Dipeptide Derivative
UDP-glucose	C00029	HMDB00286	8629	**0.70**	Carbohydrate	Nucleotide Sugar
sorbitol	C00794	HMDB00247	5780	**0.68**	Carbohydrate	Fructose, Mannose and Galactose Metabolism
N-acetylarginine	C02562	HMDB04620	67427	**0.63**	Amino Acid	Urea cycle; Arginine and Proline Metabolism
maltotriose	C01835	HMDB01262	439586	**0.62**	Carbohydrate	Glycogen Metabolism
oleoylcarnitine	--	HMDB05065	6441392;53477789	**0.60**	Lipid	Fatty Acid Metabolism(Acyl Carnitine)
maltose	C00208	HMDB00163	10991489	**0.54**	Carbohydrate	Glycogen Metabolism
N6-carboxymethyllysine	--	--	--	**0.52**	Carbohydrate	Advanced Glycation End-product
linoleoylcarnitine*	--	HMDB06469	6450015	**0.52**	Lipid	Fatty Acid Metabolism(Acyl Carnitine)
N2-acetyllysine	C12989	HMDB00446	92907	**0.38**	Amino Acid	Lysine Metabolism

Table S3: Metabolites that were decreased ($p < 0.05$) in Ric mKO compared to Ric WT muscle ($n = 8$).
